# |Isolation and characterization of novel bacteriophages as a potential therapeutic option for *Escherichia coli* urinary tract infections

**DOI:** 10.1007/s00253-021-11432-6

**Published:** 2021-07-12

**Authors:** Edgar González-Villalobos, Rosa María Ribas-Aparicio, Gerardo Erbey Rodea Montealegre, Laura Belmont-Monroy, Yerisaidy Ortega-García, Gerardo Aparicio-Ozores, José Luis Balcázar, Carlos Alberto Eslava-Campos, Ulises Hernández-Chiñas, José Molina-López

**Affiliations:** 1 Departamento de Microbiología, Escuela Nacional de Ciencias Biológicas (ENCB), Instituto Politécnico Nacional (IPN), Prolongación de Carpio y Plan de Ayala s/n, Colonia Santo Tomás, C.P. 11340 Mexico City, Mexico; 2 Unidad Periférica de Investigación Básica y Clínica en Enfermedades Infecciosas, Departamento de Salud Pública/División de Investigación, Facultad de Medicina, UNAM, C.P. 04510 Mexico City, Mexico; 3 Laboratorio de Patogenicidad Bacteriana, Unidad de Hemato-Oncología e Investigación, Hospital Infantil de México Federico Gómez/Facultad de Medicina, UNAM, Dr. Márquez 162 Col. Doctores. Alcaldía Cuauhtémoc, C.P. 06720 Mexico City, Mexico; 4 Catalan Institute for Water Research (ICRA), 17003 Girona, Spain; 5 University of Girona, 17004 Girona, Spain

**Keywords:** Adherence, Biofilm, Multidrug resistance, Phage therapy, Urinary tract infections

## Abstract

**Abstract:**

Urinary tract infections (UTIs) are mainly caused by uropathogenic *Escherichia coli* (UPEC), whose impact can be exacerbated by multidrug-resistant (MDR) strains. Effective control strategies are, therefore, urgently needed. Among them, phage therapy represents a suitable alternative. Here, we describe the isolation and characterization of novel phages from wastewater samples, as well as their lytic activity against biofilm and adherence of UPEC to HEp-2 cells. The results demonstrated that phage vB_EcoM-phiEc1 (ϕEc1) belongs to *Myoviridae* family, whereas vB_EcoS-phiEc3 (ϕEc3) and vB_EcoS-phiEc4 (ϕEc4) belong to *Siphoviridae* family. Phages showed lytic activity against UPEC and gut commensal strains. Phage ϕEc1 lysed UPEC serogroups, whereas phages ϕEc3 and ϕEc4 lysed only UTI strains with higher prevalence toward the O25 serogroup. Moreover, phages ϕEc1 and ϕEc3 decreased both biofilm formation and adherence, whereas ϕEc4 was able to decrease adherence but not biofilm formation. In conclusion, these novel phages showed the ability to decrease biofilm and bacterial adherence, making them promising candidates for effective adjuvant treatment against UTIs caused by MDR UPEC strains.

**Key points:**

- *Phage with lytic activity against MDR UPEC strains were isolated and characterized under in vitro conditions.*
- *A novel method was proposed to evaluate phage activity against bacterial adherence in HEp-2 cell.*.
- *Phages represent a suitable strategy to control infections caused by MDR bacteria*.

**Supplementary Information:**

The online version contains supplementary material available at 10.1007/s00253-021-11432-6.

## Introduction

Urinary tract infections (UTIs) are one of the most common health problems, affecting 150 million of people each year worldwide (Tamadonfar et al. 2019). In 2007, there were an estimated 10.5 million ambulatory visits for UTI symptoms and 2–3 million emergency department visits in the USA (Foxman 2010, 2014), reaching a cost of more than USD 6 billion on its control and treatment (Mann et al. 2017). Community-acquired UTI is the most frequent infection, whose main etiological agent is *Escherichia coli*. UTIs are usually caused by uropathogenic *E*. *coli* (UPEC), with frequencies around 95% for community-acquired and 80% for uncomplicated UTIs in both inpatients and outpatients (Kot 2019). Among them, UPEC serogroups O1, O2, O4, O6, O7, O8, O15, O16, O18, O21, O22, O25, 075, and O83 have been associated more frequently with UTIs (Li et al. 2010).

UPEC pathogenesis includes two main phases: bacterial adherence to urogenital epithelium and biofilm production. In the first phase, UPEC binds to glycosylated surface proteins (uroplakins) using type 1 fimbriae. This binding leads to invasion of bladder epithelial cells by UPEC, which can escape into the cytoplasm and form intracellular bacterial communities (IBCs) (Terlizzi et al. 2017). In the second phase, biofilm-producing microorganisms such as UPEC show high resistance to antibiotics, preventing the arrival of antibiotics at the target site. Additionally, biofilm formation allows bacteria to resist the normal urine flow, favoring persistence in the urinary tract (Subaschandrabose and Mobley 2015; Magana et al. 2018). Biofilm production also enhances multidrug-resistance (MDR) phenomenon (Hall and Mah 2017; Karigoudar et al. 2019). MDR pathogenic bacteria are a growing global problem that represents a large clinical and public health burden, as the number of emerging bacterial pathogens resistant to available antibiotics rapidly increases. In fact, the World Health Organization has recently listed UPEC as a critical priority pathogen (Tacconelli and Magrini 2020).

The global emergence of MDR pathogenic bacteria is driving the need for research into effective therapeutic antimicrobial alternatives. Bacteriophage therapy represents a promising alternative to antibiotics, which is based on the bactericidal activity of phages (Gordillo-Altamirano and Barr 2019). Phages recognize receptors on the bacterial surface with high specificity, inject their genetic material, multiply and assemble inside the bacterium, to finally break it and release their progeny to infect new bacteria (Jamal et al. 2019). The worldwide antibiotic crisis has led to a renewed interest in phage therapy. Phages with strong lytic activity against bacterial pathogens can be isolated from the environment. In addition, phages have the capacity to rapidly overcome bacterial resistance, which will inevitably emerge (Principi et al. 2019).

Given the clinical importance of UTIs, the aim of this work was to identify new phages with lytic features against UPEC. Here, we report an analysis of the characteristics of three phages isolated from wastewater samples in Mexico City (Mexico), considering both the ability to eliminate bacteria adhered to HEp-2 cells and decrease biofilm formation and their lytic activity against UPEC strains causing acute and persistent UTIs.

## Methods

### Bacterial isolates

One hundred fifty MDR *E*. *coli* isolates, previously serotyped and characterized by MDR patterns, were used in this study (Ahumada-Cota et al. 2020). The strains were initially grouped according to their origin: 50 strains isolated from stool cultures, 50 strains from recurrent UTIs at the “Federico Gómez” Children’s Hospital (Mexico City), and 50 strains from acute UTIs at the Family Medicine Unit No. 61 of the Mexican Social Security Institute (IMSS). All strains are available in our collection, which belongs to the World Data Centre for Microorganisms (WDCM 449).

### Bacterial biofilm production

Each *E*. *coli* isolates was screened for biofilm formation using a 24-well plate assay, as previously described (Chibeu et al. 2012). Briefly, *E*. *coli* isolates were inoculated in 3 mL of Luria-Bertani (LB) broth and incubated at 37 °C for 18 h. Immediately after, bacterial suspensions were adjusted to 1.5 × 10^8^ CFU mL^−1^, ten-fold diluted using minimum essential medium (MEM) supplemented with 1% glucose, and 450 μL of this suspension was added into a 24-well plate (Corning Inc., Corning, NY, USA). The plate was incubated at 37 °C for 24 h without agitation. After the incubation period, the plate was washed twice with 1×PBS to remove free bacteria, 1% crystal violet (w/v) was then added to stain the biofilm mass and incubated at room temperature for 15 min. Crystal violet was removed by two washes with deionized water; the plates were dried at room temperature for 10 min, and stained biofilm was solubilized with the addition of 200 μL ethanol 96% (w/v). The absorbance was then measured at an optical density of 590 nm using an Epoch microplate reader (BioTek, Winooski, VT, USA). The results were obtained by taking the average from three replicates in three independent assays. *E*. *coli* K-12 and *E*. *coli* 49766 were used as negative and positive controls, respectively. The strains were classified as weak, moderate, and strong biofilm producers, according to Stepanović et al. 2007.

### Assays of bacterial adherence in HEp-2 cells

HEp-2 cells were grown in a 24-well tissue culture plate on circular 13-mm glass coverslips in MEM without fetal bovine serum, until an 80–90% confluence was obtained. The tested strains were grown overnight in tryptone water with 1% d-mannose to inhibit type 1 fimbriae-mediated attachment, and 1 × 10^7^ CFU mL^−1^ of this culture was then added into each well. The plates were incubated at 37 °C with 5% CO_2_ for 3 h. At the end of infection, cells were washed three times with 1×PBS and fixed with methanol for 15 min, stained with 1% Giemsa for 20 min, and examined under optical microscope (Mathewson and Cravioto 1989). *E*. *coli* K-12 and *E*. *coli* 49766 were used as negative and positive controls, respectively, and the adhered bacteria were counted microscopically in at least 15 fields.

### Phage isolation

Six wastewater samples were collected from Mexico City, which were centrifuged, and the supernatant filtered through 0.22-μm pore-size to remove any bacteria present in the samples. These filtered samples were used to isolate phages, in which 500 μL of each was added to a mid-log phase of different *E*. *coli* strains (*E*. *coli* K-12, *E*. *coli* ATCC 25922, *E*. *coli* CFT073 and two MDR *E*. *coli* isolates from UTI). The infected culture was incubated at 37 °C with shaking for 24 h and then tested for plaque formation using the double-layer agar method, as previously described (Kropinsky et al. 2009). Lytic plaques were selected and isolated for propagation and further evaluation. These phages have been deposited and are available in our collection, which belongs to the World Data Centre for Microorganisms (WDCM 449).

### Phage purification

Only three phages were selected and propagated in the mid-log phase of the permissive strain. Cell debris and non-lysed cells were then removed by centrifugation at 10,000 *g* for 20 min and the supernatant was filtered through 0.22-μm pore-size filter, which was recovered and incubated at 37 °C in presence of RNase and DNase (1 μg mL^−1^ and 2 U, respectively) for 30 min to remove any residual bacterial DNA, as previously suggested (Zhao et al. 2019). NaCl was then added to reach 1 M, mixed, incubated at 4 °C for 1 h, and subsequently centrifuged at 10,000 *g* for 20 min. The supernatant was recovered and PEG 8000 was added (10%; [w/v]) and incubated overnight at 4 °C, followed by a chloroform extraction. The aqueous phase was ultracentrifuged at 500,000 *g* for 2 h using a caesium chloride density gradient, and the recovered fraction was dialyzed with deionized water (Sambrook and Russell 2001). The phage particles were quantified using the small drop plaque assay, as previously described (Mazzocco et al. 2009).

### Phage morphology

A volume of 10 μL of a purified phage suspension (1 × 10^8^ PFU mL^−1^) was placed on a Formvar-carbon coated grid for 2 min, which were negative-stained with 5 μL of 1% phosphotungstic acid for 2 min (Hans 2009), and the morphology was visualized under a JEM-1010 transmission electron microscope (JEOL; Tokyo, Japan) at the Central Laboratory for Microscopy Instrumentation, Escuela Nacional de Ciencias Biológicas, Instituto Politécnico Nacional.

### Isolation of genomic phage DNA

Phage DNA was obtained from purified phage particles (1 × 10^13^ PFU mL^−1^), as previously described (Sambrook and Russell 2001). Briefly, proteinase K (final concentration at 50 μg mL^−1^) and 10% SDS were added and incubated at 56 °C for 1 h. DNA was then extracted with phenol:chloroform (1:1) and ethanol (−   70 °C) precipitation, resuspended in deionized water, and quantified spectrophotometrically using an Epoch microplate reader (BioTek). Later, 1 μg of the purified DNA was digested with restriction enzymes such as *Ssp*I, *Eco*RI, *Eco*RV, and *Eco*RI-*Eco*Rv (New England Biolabs, Ipswich, MA, USA), following manufacturer’s recommendations. The products obtained were resolved by electrophoresis on 1% agarose gels.

### SDS-PAGE

Proteins of selected phages were identified according to the procedure previously described (Boulanger 2009). Briefly, the phages were precipitated with PEG/NaCl, equal volumes of phage solution and LiCl (10 M) were mixed and incubated at 46 °C for 30 min. DNase (50 U) was added and incubated at 37 °C for 2 h. Phage particles were concentrated at 500,000 *g* at 4 °C for 30 min using a Sorvall ultra centrifuge. Concentrated phages were mixed with 2× Laemmli solution (65.8 mM Tris–HCl [pH 6.8], 2.1% SDS, 26.3% glycerol [w/v], 0.01% bromophenol blue and 100 mM β-mercaptoethanol) and heated at 100 °C for 10 min. Proteins were then separated by 15 % SDS-PAGE (polyacrylamide-sodium dodecyl sulfate gel electrophoresis) and visualized using Coomassie blue or silver staining.

### Image analysis, spot excision, destaining, and drying

Individually resolved bands were excised with an EXQuest Spot Cutter (Bio-Rad; Hercules, CA, USA) and processed following Bruker’s standard protocol for in-gel protein digestion with minor modifications. Briefly, gel particles were washed three times with 50 mM NH_4_HCO_3_ and CH_3_CN mixed in equal proportion. The proteins were reduced with 10 mM DTT (at 56 °C for 45 min) and alkylated with 55 mM C_2_H_4_INO (at room temperature in the dark for 20 min). Samples were then washed again with 50 mM NH_4_HCO_3_ and CH_3_CN, the supernatant was removed, and the gel particles were air-dried.

### Trypsin digestion, peptide extraction, and MALDI-TOF/TOF

For digestion of the proteins, the gel particles were incubated with 25 ng/L trypsin gold (Promega; Madison, WI, USA) in 25 mM NH_4_HCO_3_ at 37 °C overnight. The supernatants were recovered and stored at −   20 °C, whereas the gel particles were incubated a second time with 50 mM NH_4_HCO_3_ at 37 °C overnight and the supernatants were then stored at −   20 °C. Peptides were extracted in 50 μL trifluoroacetic acid 0.1%/acetonitrile (1:1) for 30 min at room temperature. All supernatants were mixed and dried in a vacuum centrifuge. Peptides were resuspended in trifluoroacetic acid and analyzed using a MALDI-TOF (MS/MS) Ultraflextreme mass spectrometer (Bruker; Billerica, MA, USA). An AnchorChip target and α-cyano-4-hydroxycinnamic acid (HCCA) matrix were used, according to the manufacturer’s instructions. The laser intensity was adjusted to 50% for the acquisition of masses, with three or four repetitions. For the analysis, spectra with 1 × 10^3^–1 × 10^4^ intensity peaks were considered, and the identity of the protein was matched using the Mascot search engine with the following parameters: enzyme, trypsin; missed cleavages, 1; fixed modification, carbamidomethyl C; variable modification, oxidation M; parent tolerance, 0.2 Da; fragment tolerance, 0.5 Da. Positive protein identifications were considered reliable with a Mascot score higher than 30.

### Phage-host range assays

The host range of isolated phages was performed according to the previously described spot test (Kutter 2009). Briefly, each *E*. *coli* isolate was grown until the mid-log phase, and 200 μL were mixed with 3 mL molten agar and poured on LB plates. A spot of 10 μL of each phage (final concentration at 1 × 10^6^ PFU mL^−1^) was added and left dry, and the plates were then incubated at 37 °C for 24 h. Phage specificity was also tested on the following strains: *Salmonella enterica* subsp. *enterica* serovar Typhi ATCC 6539, *Salmonella enterica* subsp. *enterica* serovar Choleraesuis ATCC 10708, *Salmonella enterica* subsp. *enterica* serovar Typhimurium ATCC 14028, *Pseudomonas aeruginosa* ATCC 9027, and clinical isolates of *Yersinia enterocolitica*, *Klebsiella pneumoniae*, *Shigella boydii*, and *Staphylococcus aureus*, in which the spot test was used, as previously described (Mazzocco et al. 2009).

### Effect of the phages on biofilm

Twenty-one biofilm-forming strains were selected based on their susceptibility to isolated phages and biofilm production. Briefly, 1 × 10^7^ PFU mL^−1^ of the selected phage was added into biofilm, previously grown for 18 h, and the plate was then incubated for 1 h. After the incubation period, the plate was washed twice with 1×PBS, and the remaining biofilm was stained and evaluated, as previously described (Chibeu et al. 2012).

### Effect of the phage infection on bacterial adherence

Eighteen isolates were selected based on phage susceptibility and adherence assay. We designed the adherence challenge at the end of conventional infection assay as follows: cells were washed with 1×PBS to remove any unattached bacteria, then 1 × 10^6^ PFU mL^−1^ of each phage was added to each well and the plates were incubated at 37 °C with 5% CO_2_ for 2 h. The remaining of the assay was completed as previously described (Mathewson and Cravioto 1989). The phages were recovered and quantified after assay, which were performed in duplicate. HEp-2 cell was also incubated with phages to discard damage on the monolayer.

### Statistical analyses

All statistical analyses were performed using GraphPad Prism software version 8.00 (GraphPad Software Inc., San Diego, CA, USA). The effect of phages on biofilm was analyzed using the unpaired Student’s *t* test, whereas its effect on UPEC attached to HEp-2 cells was analyzed using analysis of variance (ANOVA). Differences were considered statistically significant at *P* < 0.05.

## Results

### Isolation and morphology of selected phages

**Fig. 1:**
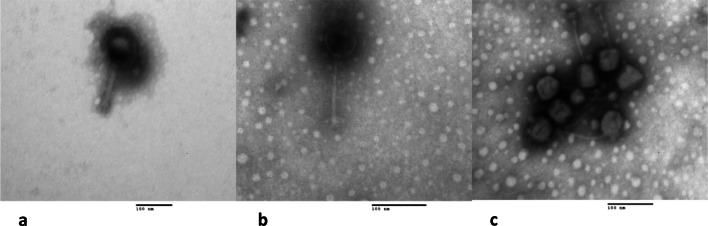
Morphology of phages ϕEc1, ϕEc3 and ϕEc4. Transmission electron micrographs of phages ϕEc1 (**a**), ϕEc3 (**b**), and ϕEc4 (**c**)

Phages were isolated from six different wastewater locations in Mexico City. Due to the plaque morphologies, only three phages were further characterized (data not shown), which were named vB_EcoM-phiEc1 (ϕEc1), vB_EcoS-phiEc3 (ϕEc3), and vB_EcoS-phiEc4 (ϕEc4), according to the current naming conventions (Adriaenssens and Brister 2017). Electron microscopy revealed different phage morphological features (Fig. 1). Phage ϕEc1 showed an icosahedral head of 76 nm in diameter and a contractile tail of 126 nm in length (Fig. 1a), whereas phages ϕEc3 and ϕEc4 exhibited an icosahedral head of 60 nm in diameter and a long non-contractile tail with six fibers (Fig. 1 b and c).

### Lytic activity against UPEC and phage host-range

Lytic activity of phages ϕEc1, ϕEc3, and ϕEc4 was tested against three groups of *E*. *coli* strains (acute UTI, recurrent UTI, and stool cultures) using the spot test. Phage ϕEc1 showed the broadest host range, lysing 35/150 (23.3%) strains. Among them, 18/35 (51.4%) acute UTI strains, 10/35 (28.5%) strains from stool cultures, and 7/35 (20%) recurrent UTI strains exhibited susceptibility to phage ϕEc1. Phage ϕEc3 also showed the ability to lyse 18/150 (12%) strains, of which 7/18 (38.8%) acute UTI strains were lysed, followed by 11/18 (61.1%) strains belonging to recurrent UTI; however, no effect was detected against strains from stool cultures. Moreover, phage ϕEc4 showed the ability to lyse 12/150 (8%) strains, of which 7/12 (58.3%) acute UTI strains were lysed, followed by 5/12 (41.6%) recurrent UTI strains; however, phage ϕEc4 did not lyse strains from stool cultures.

Additionally, the host range of phages ϕEc1, ϕEc3, and ϕEc4 was tested against the following bacterial species: *S*. *enterica* subsp. *enterica* serovar Typhi, *S*. *enterica* subsp. *enterica* serovar Choleraesuis, *S. enterica* subsp. *enterica* serovar Typhimurium, *Pseudomonas aeruginosa*, *Yersinia enterocolitica*, *Klebsiella pneumoniae*, *Shigella boydii*, *Staphylococcus aureus*, and *Escherichia coli* (ATCC 25966, K-12, CFT073, and 49766 strains). All tested strains were resistant to phages ϕEc1, ϕEc3, and ϕEc4.

### Enzymatic analysis of selected phages

**Fig. 2:**
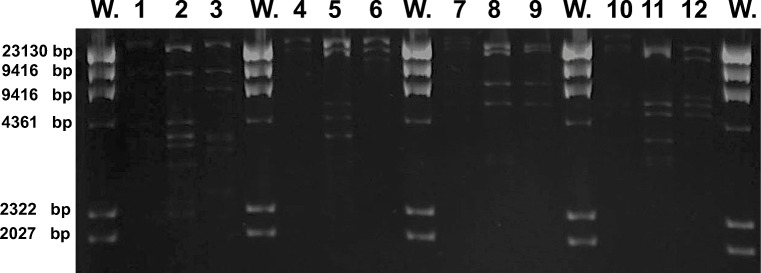
Restriction analysis of genomic ϕEc1, ϕEc3, and ϕEc4 DNA. Molecular-weight size marker (lane W). Total DNA of ϕEc1, ϕEc3, and ϕEc4 digested with *Ssp*I (lanes 1–3), *Eco*RI (lanes 4–6), *Eco*RV (lanes 7–9), and *Eco*RI-*Eco*RV (lanes 10–12), respectively

Genomic differences were determined by DNA restriction, and total phage DNA was thus digested with *Ssp*I, *Eco*RI, *Eco*RV, and *Eco*RI-*Eco*RV (Fig. 2). Restriction with *Ssp*I showed differences among the three phages. Although all phages shared common fragments, the restriction pattern of phage ϕEc1 was different than the remaining phages. Phage ϕEc3 and ϕEc4 shared a common pattern; however, phage ϕEc3 possess 3 unique fragments (Fig. 2, lanes 1–3). Restriction with *Eco*RI showed a unique pattern for phage ϕEc3 (Fig. 2, lane 5), whereas restriction with *Eco*RV showed common fragments among them (Fig. 2, lanes 7, 8, 9). However, phage ϕEc3 showed additional unique fragments (Fig. 2, lane 8). Although restriction patterns generated with *Eco*RI-*Eco*RV revealed common fragments among them (Fig. 2, lanes 10, 11, 12), phage ϕEc3 showed additional unique fragments (Fig. 2, lane 11).

### Analysis of protein profiles

**Fig. 3:**
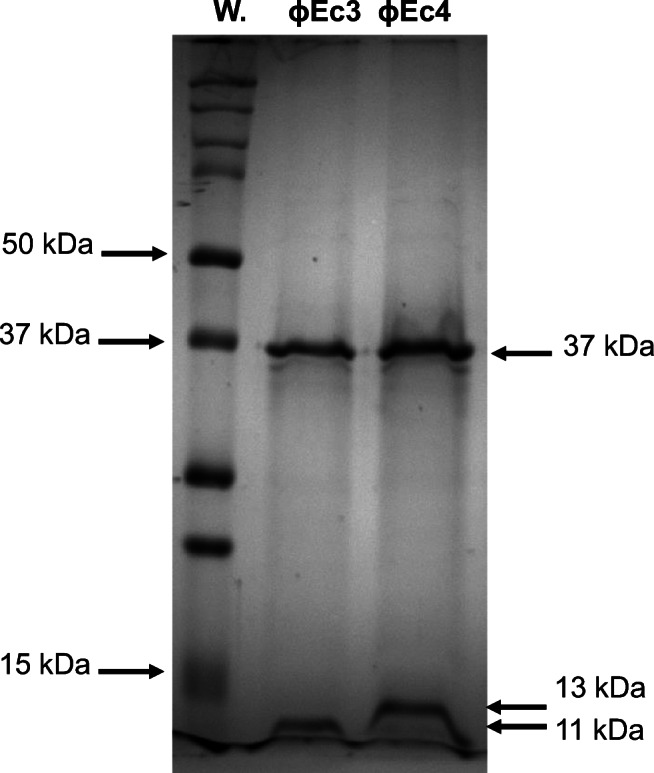
Analysis of ϕEc3 and ϕEc4 protein profiles (15% SDS-PAGE stained with Coomassie blue). ϕEc3, two proteins of 37 kDa and 11 kDa were identified. ϕEc4, two proteins of 37 kDa and 13 kDa were identified

To identify proteins, phage lysates were resolved on 15% SDS-PAGE gel electrophoresis. A protein of approximately 50 kDa was detected in phage ϕEc1 (data not shown), whereas two proteins were detected in phage ϕEc3 (the first one has a molecular weight of approximately 36–37 kDa and the second one of approximately 11 kDa). Two proteins were also detected in phage ϕEc4 (one of 36–37 kDa and another of approximately 13 kDa). Similar staining profiles were observed with Coomassie blue and silver staining (Fig. 3 and Fig. S1). The heavier protein was not size-related between ϕEc1 and ϕEc3 or ϕEc4, nor the smaller protein between them. According to mass spectrometry data, the higher molecular weight (MW) proteins corresponded to RNA2 polyprotein and endolysin (Table S1), whereas the lower MW proteins showed an average of 25 m/z peaks which did not show significant matches in Mascot.

### Bacterial biofilm production and effect of selected phages

One hundred-fifty *E*. *coli* were examined for biofilm production, and only 48 (32%) of the 150 isolates were biofilm producers. Predominant biofilm phenotype was weak, with 28 strains followed by 16 moderate and only 4 strong producers. The majority biofilm producer strains belonged to isolates from stool cultures 19 (12.7%), followed by recurrent UTI with 15 (10%), and finally acute UTI with 14 (9.3%).

**Fig. 4:**
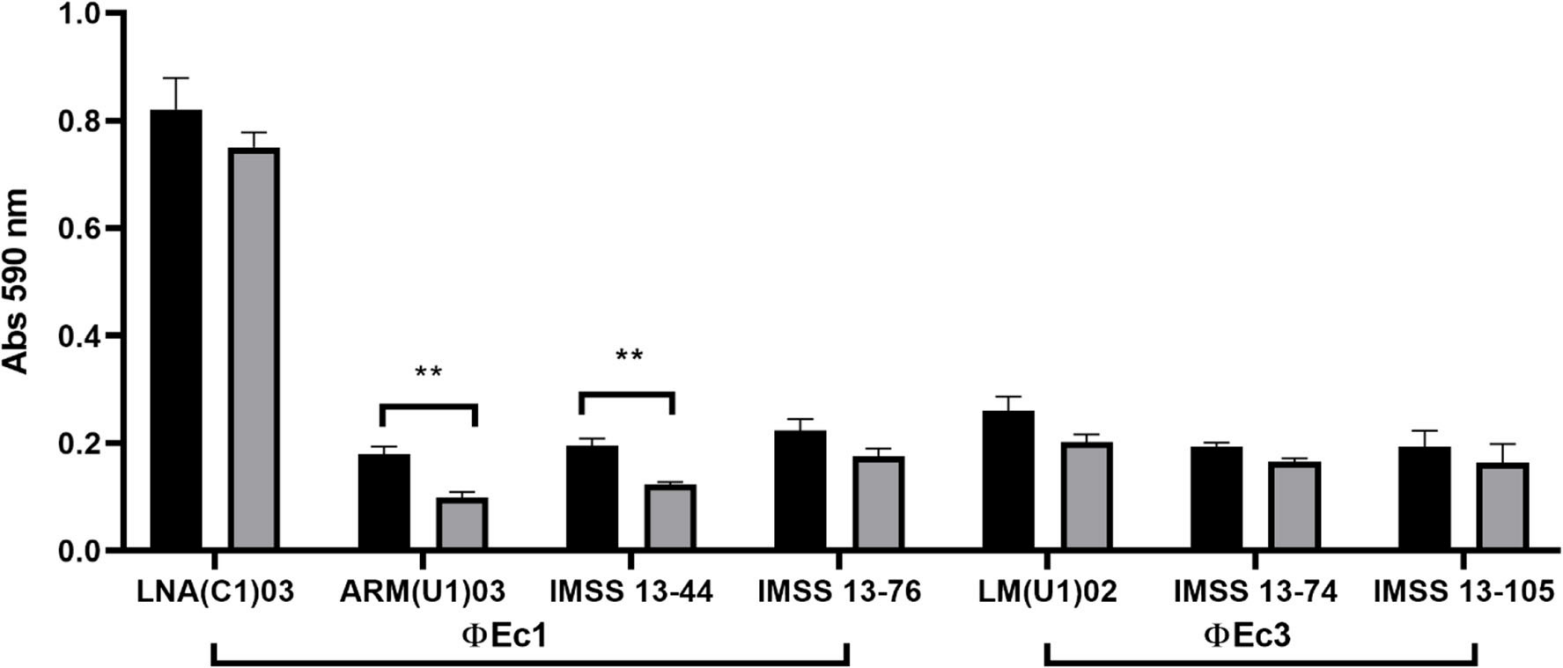
Phage effect on biofilm production. Biofilm was decreased after 1 h of exposure. Statistical differences were observed after incubation with phage ϕEc1 (*p* = 0.01), whose values were assessed and calculated using Student’s t-test with GraphPad Prism 8. Bars represent SEM

Twenty-one biofilm-forming strains were selected based on the phage susceptibility in order to analyze the effect of the isolated phages on them. Some of the selected strains were susceptible to at least one phage. In these cases, the strains were then tested with each of them. Phage ϕEc1 decreased biofilm formation in 4/9 strains, but a statistical difference was only observed in two cases (Fig. 4). On the other hand, phage ϕEc3 was able to decrease biofilm in 3/6 of the strains tested (Fig. 4), whereas phage ϕEc4 had no effect on biofilm formation (data not shown).

### Relationship between serogroup and phage susceptibility

The isolated phages showed activity against strains from UPEC serogroups, as well as from non-UPEC serogroups (Table 1). Phage ϕEc1 showed lytic activity against strains belonging to non-UPEC serogroups, whereas phages ϕEc3 and ϕEc4 showed lytic activity against bacteria belonging to UPEC serogroups, mostly of the O25 serogroup.

**Table 1:** Relationship between the production of biofilm, adherence, serogroup, and susceptibility of *E*. *coli* strains to phage infection

	Strain	Origin	Serogroup	Biofilm	Adherence	Phage susceptibility		
						ϕEc1	ϕEc3	ϕEc4
UPEC serogroups	CMP(U1)01	RUTI	O25	−	+	−	+	+
	GPB(U2)03		O25	−	−	−	+	+
	LM(U1)02		O25	Weak	+	−	+	+
	RMO(U1)07		O25	Weak	+	−	+	−
	RMR(U5)05		O25	Weak	D/C	−	−	+
	PSS(U1)09		O6	Moderate	D/C	+	−	−
	IMSS 13-20	aUTI	O25	Moderate	+	−	+	+
	IMSS 13-58		O25	−	+	+	−	−
	IMSS 13-64		O25	−	+	+	−	−
	IMSS 13-68		O25	−	D/C	−	+	−
	IMSS 13-81		O25	−	D/C	−	+	+
	IMSS 13-91		O25	−	−	+	−	−
	IMSS 13-101		O25	Weak	−	+	−	−
	IMSS 13-105		O25	Weak	−	−	+	+
	IMSS 13-119		O25	Strong	+	+	−	−
	IMSS 13-39		O75	−	−	−	−	+
	IMSS 13-43		O75	−	D/C	−	+	+
	IMSS 13-55		O75	−	−	+	−	−
	IMSS 13-44		O6	Weak	−	+	−	−
	IMSS 13-15		O8	−	+	−	+	+
NON UPEC serogroups	AA(C1)02	COM	NO UPEC	−	−	+	−	−
	CMP(C2)04		NO UPEC	Moderate	−	+	−	−
	CMV(C1)08		NO UPEC	Weak	−	+	−	−
	LNA(C1)03		NO UPEC	Weak	D/C	+	−	−
	LNA(C1)07		NO UPEC	−	−	+	−	−
	PSL(C1)05		NO UPEC	−	−	+	−	−
	RMR(C1)04		NO UPEC	−	D/C	+	−	−
	IMSS 13-4	aUTI	NO UPEC	−	−	+	−	−
	IMSS 13-5		NO UPEC	−	−	+	−	−
	IMSS 13-22		NO UPEC	−	−	+	−	−
	IMSS 13-48		NO UPEC	Weak	+	+	−	−
	IMSS 13-49		NO UPEC	−	+	+	−	−
	IMSS 13-74		NO UPEC	Weak	D/C	−	+	+
	IMSS 13-76		NO UPEC	Weak	+	+	−	−
	IMSS 13-93		NO UPEC	−	−	+	−	−
	IMSS 13-97		NO UPEC	−	−	+	−	−
	IMSS 13-99		NO UPEC	−	−	+	−	−
	IMSS 13-100		NO UPEC	Weak	−	+	−	−
	IMSS 13-134		NO UPEC	Weak	+	+	−	−
Non typeable	ARM(U2)06	RUTI	NT	Weak	+			
	EC(U1)02		NT	−	D/C			
	EC(U1)07		NT	Moderate	D/C			
	EGR(U1)05		NT	Weak	D/C			
	GPB(U3)09		NT	−	−			
	MCB(U1)01		NT	−	+			
	OGO(U2)05		NT	−	−			
	XZG(U2)01		NT	−	+			
	S(C1)01	COM	NT	−	+	+	−	−
	TCJ(C1)04		NT	−	+	+	−	−
	TCJ(C1)08		NT	−	+	+	−	−
OR	ARM(U1)01	RUTI	OR	−	−	−	+	−
	ARM(U1)03		OR	Weak	−	+	+	−
	CMV(U1)03		OR	−	−	+	−	−

RUTI: *E*. *coli* isolates from recurrent UTI; aUTI: *E*. *coli* isolate from acute UTI; COM: gut commensal strain; NT: Non Typeable, OR: rough strain, D/C: bacteria promoted the detachment of the monolayer HEp-2 cells

### Bacterial adherence in HEp-2 cells

Eighty-three (55.3%) strains were adherent to HEp-2 cells. Adherence frequencies were found to be very similar among the groups, with 20.7% for strains isolated from stool, followed by strains from acute and recurrent UTI with 17.3% each. The number of adhered bacteria was at least 5 per cell, in which positive strains showed patterns of aggregative and diffuse adherence. No relationship was observed between adherence ability and strain origin.

**Fig. 5:**
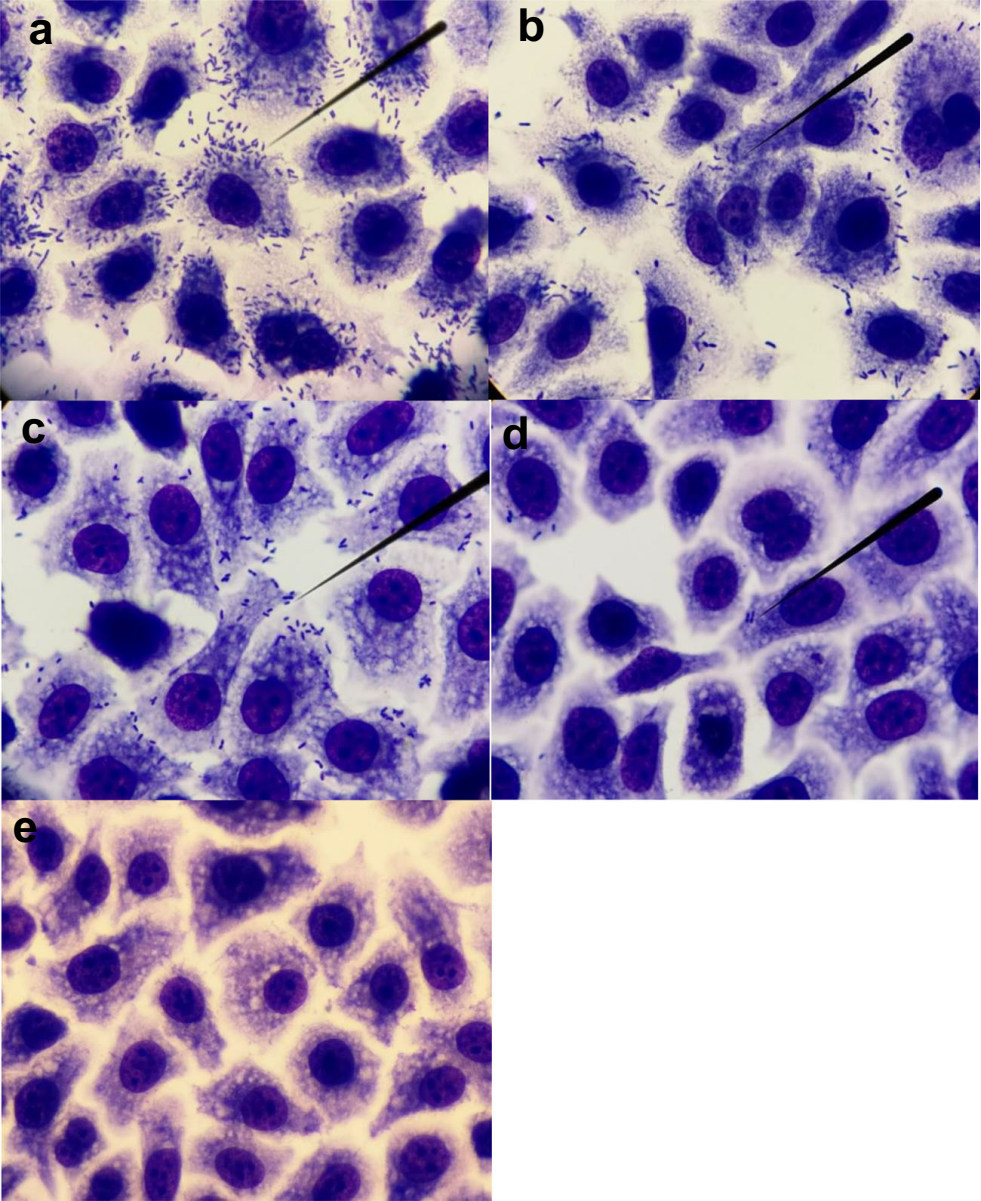
Phage effect on bacterial adherence. UPEC adherence was decreased after 1 h of phage infection. Effect of phage ϕEc1: **a** control assay HEp-2 cells-UPEC, **b** bacterial adherence after phage exposure. Effect of phage ϕEc3: **c** bacterial adherence without phages, **d** phage ϕEc3 almost eliminated all attached bacteria. **e** Control Hep-2 cells

**Fig. 6:**
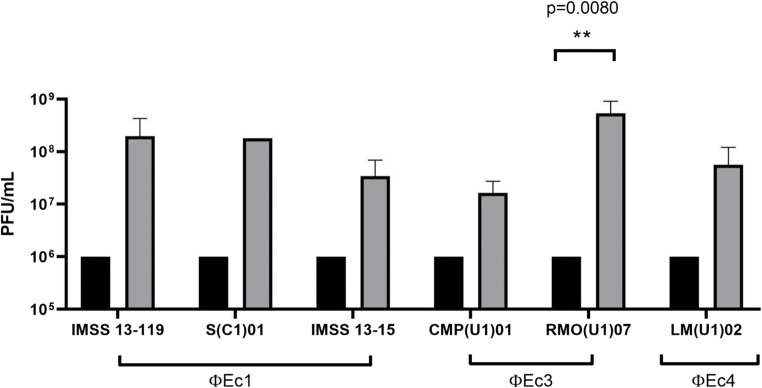
Phages effect on UPEC attached to HEp-2 cells. Phage titers before and after UPEC infection. Higher PFU numbers were recovered after initial *E*. *coli* infection, whose values were assessed and calculated using ANOVA with GraphPad Prism 8. Bars represent SEM

Eighteen strains with positive adherence and susceptibility to phage infection were selected, in order to assess the effect of phages on bacterial adherence. Phage ϕEc1 and ϕEc3 decreased the adherence in two and three strains, respectively, but phage ϕEc4 was only effective for one strain (Fig. 5). In order to ensure that adherence reduction was a consequence of phage infection, the phage suspension was adjusted to 1 × 10^6^ PFU mL^−1^ at the beginning of the assay. After 2 h of exposure, phages were quantified and their titers showed an increase of at least 1 log (Fig. 6), which was consistent in all microscopic observations performed after phage infection.

## Discussion

As we live in the antibiotic era, it was easy to assume that these agents would be permanently available in the drug arsenal. However, the overuse and misuse of antibiotics has been accompanied by the rapid emergence of antibiotic-resistant bacteria (Morehead and Scarbrough 2018). Antibiotic resistance is life-threatening, which raises mortality rates, increases human suffering, extends hospitalization periods, and decreases productivity, along with the economic burden that takes a staggering toll in the health care system (Santoro et al. 2020; Serra-Burriel et al. 2020).

The worldwide emergence of MDR bacterial strains has created the need for implementing measures to control these threats. As a consequence, lytic phages have reemerged as a promising alternative for the control of pathogenic bacteria (Kakasis and Panitsa 2019; Ghosh et al. 2019). Because phage therapy is under study as a therapeutic approach, further development of this method requires biological characterization of phages, such as their host specificity and adaptation to their bacterial hosts (Amarillas et al. 2017; Sváb et al. 2018; Hyman 2019).

From the six wastewater sample locations, 12 phages were originally isolated, and only three were further characterized. Selected phages displayed the basic features to classify them into two different morphologies. The morphology of phage ϕEc1 resembles the *Myoviridae* family, distinguished by an extraordinarily large icosahedral head, which contracts to half of its original length upon infection. In contrast, the morphology of phages ϕEc3 and ϕEc4 resemble the *Siphoviridae* family, whose members have icosahedral heads and non-contractile and flexible tails (King et al. 2012). Previous studies have demonstrated that the *E*. *coli*-infecting phages usually belong to the *Myoviridae* family. However, a recent study demonstrated that *E*. *coli*-infecting phages, previously isolated from Danish wastewater, belonged to seven different families, such as *Myoviridae*, *Siphoviridae*, *Podoviridae*, *Drexlerviridae*, *Chaseviridae*, *Autographviridae*, and *Microviridae* (Olsen et al. 2020). Despite the wide diversity of *E*. *coli*-infecting phages, many studies are restricted to the isolation and characterization of phages for food industry applications (Khalatbari-Limaki et al. 2020). Moreover, limited information is available on the use of phages against MDR UPEC strains. In this study, the three phages showed specificity for *E*. *coli* clinical isolates, as no infectivity was detected for *Salmonella*, *Pseudomonas*, *Yersinia*, *Klebsiella*, *Shigella*, and *Staphylococcus*.

Phage ϕEc1 showed a broader range through the *E*. *coli* strain groups (acute, recurrent, and stool cultures). Likewise, phage ϕEc1 lysed 23% (35/150) of clinical isolates and the group with the highest susceptibility was acute strains with 51% (18/35). Most of the lysed strains belonged to non-UPEC serogroups including non-typeable (NT) and “O” rouge (OR) strains. Although phages ϕEc3 and ϕEc4 lysed fewer strains than did ϕEc1, their effect was restricted to UTI (acute and recurrent) strains. Specifically, the serogroup sensitive to the phages wasO25, which is one of the most frequently isolated serogroups (17%) in Mexico, followed by O1 (10%), O8 (9%), O6 (4%), and O75 (3%) (Belmont-Monroy et al. 2017). These serogroups have also been associated up to 60% with extensively drug-resistant *E*. *coli* due to the production of extended-spectrum β-lactamases (Hernández-Chiñas et al. 2019). Therefore, phages ϕEc3 and ϕEc4 with specific UPEC spectrum can be useful for therapy, which represent an alternative for the treatment against infections caused by virulent MDR UPEC serogroups.

Genome restriction analyses allowed us to indirectly assume genomic differences among the three phages. ϕEc1 genome showed fewer bands in all the restriction assays, which suggests its relative resistance against *E. coli* RM system (Flodman et al. 2019). This feature correlates with its broader range of infectivity and lysis against acute and recurrent UPEC strains as well as *E*. *coli* stool isolates. The patterns shown by ϕEc1 were different than those presented by the other two phages. Different fragment patterns were identified in ϕEc3 and ϕEc4 restricted genomes compared with ϕEc1. However, there are clear pattern differences between ϕEc3 and ϕEc4. Shared bands and unique bands between ϕEc3 and ϕEc4 suggest a closer phylogenetic relation between them when compared to ϕEc1 (López-Cuevas et al. 2011). Genomic restriction of both phages correlated with their narrower range against the three groups of *E. coli* tested, as both phages only infected UPEC strains.

To further demonstrate differences among phages, protein composition was analyzed by SDS-PAGE. One main protein of 50 kDa was detected in phage ϕEc1. Phages belonging to the *Myoviridae* family commonly show two major structural proteins ranging from 50 to 55 kDa. These proteins are related to the major tail sheath proteins, whereas proteins with a molecular mass of around 23–25 kDa are related to the capsid protein (O’Flaherty et al. 2004; Boulanger 2009). For phages ϕEc3 and ϕEc4, a protein of 36–37 kDa was detected. Previous studies have reported that the main proteins of the *Siphoviridae* family are found in a range from 41 to 51 kDa (Zhang et al. 2006; López-Cuevas et al. 2011). Additionally, a protein of 11 kDa was detected in ϕEc3 and 13 kDa in ϕEc4. SDS-PAGE protein analysis indicated clear composition differences among the phages, reinforcing the idea of their phylogenetic difference.

Adherence and biofilm production are key in UTI pathogenesis as both features play a crucial role in the first stages of UTIs development, and evolution toward recurrence (McLellan and Hunstad 2016). Biofilms allow *E*. *coli* to grow in a hostile environment, acting as a barrier that prevents the passage of antibiotics as well as avoiding the effect of the immune system (Hufnagel et al. 2015). The three groups of tested strains showed very low biofilm formation frequencies, with 32% (*n* = 48) of the isolates being biofilm producers. This percentage is closer to the results obtained by Behzadi et al. (2020), who isolated 250 *E*. *coli* strains from clean-catch urine samples from patients with laboratory-confirmed UTIs. The role of biofilm formation in colon-residing strains highlights its participation in the maintenance of the commensal microbiota, protecting it from the colonization of atypical bacteria (Da Re et al. 2013). It is important to note that the origin of the strains did not condition biofilm formation. Since colon-residing strains are the main reservoir for UTI development, another explanation for low biofilm production resides in sugars of the intestinal mucosa, which are essential in biofilm formation (Sicard et al. 2018).

The isolated phages decreased biofilm formation after a short exposure time; however, this effect was not evident in all the tested strains. In contrast, a previous study revealed that the effect of phage vB_EcoP-EG1 in the biofilm biomass reduction was 60% (Gu et al. 2019); however, in comparison with our study, the effect was evaluated 24 h after phage exposure. In our study, the effect can be explained due to bacterial metabolic state having been diminished during biofilm formation. Probably, this reduction does not favor the productive infection of the tested phages, and phage replication depends on the biosynthetic machinery of the host bacterium. Another point to be considered is that while there are reports of the use of depolymerases by phages belonging to the order of the *Caudovirales* (Fernandes and São-José 2018; Liu et al. 2019), the products derived from bacterial lysis stimulate the increase of a crystalline matrix and as a result increase adherence and resistance to desiccation (Secor et al. 2015). In order to improve biofilm study model, constant replacement of the medium could be subjected, thus eliminating the cellular remnants derived from the initial phage lysis, preventing them from being a part of the extracellular matrix.

The adherence process reflects the strategies followed by the bacterium adaptation to the different niches that it can inhabit. In the case of strains from stool cultures, it ensures the acquisition of nutrients and permanence in the intestinal tract. On the other hand, in UPEC UTI strains, it facilitates colonization and persistence in the urinary tract, as well as resistance to antimicrobials and evasion of the immune response, ensuring their survival (Conte et al. 2016; Cordeiro et al. 2016). The isolated phages showed the ability to decrease the number of bacteria in adherence assays. However, this reduction was not evident in all tested strains but only in six of them. We assume that the behavior involves factors related with the host-like metabolic state, presence of phage-receptors, or bacterial reorganization. Furthermore, there are other factors inherent to phages, such as burst size, absorption rate, and lysis time (Lindberg et al. 2014). This is the first insight exploring phage effect against UPEC adherence, and unfortunately there is no data to directly compare with our results. However, the data presented here shows a biological effect on the adhered bacteria, which showed the expected relationship of the increase in the number of phages, with the reduced number of adhered bacteria in the HEp-2 cell line.

The goal is to produce phage mixtures or identify phage proteins with a biological effect against MDR UPEC (Tagliaferri et al. 2019). However, it is important to clarify that despite the success of this trial, limitations such as the emergence of bacterial resistance after being constantly subjected to phages should not be ignored. Studies like this will expand our phage characteristics knowledge, pivotal toward in vivo studies and to ensure its safety and efficacy for human treatment (Oechslin 2018).

In conclusion, this is the first study related to phage identification in Mexico and their potential use against MDR UPEC. Although no relationship was observed between MDR and phage susceptibility, it was demonstrated that identified phages had a specific effect against *E*. *coli.* In fact, phages ϕEc3 and ϕEc4 were specific for UPECs from acute and recurrent UTIs. We also demonstrated that phages decreased biofilm formation. We highlight the phage effect against UPEC adherence on HEp-2 cells as the first report. ϕEc3 and ϕEc4 are the first phages reported in Mexico with potential use against UTIs.

## Supplementary Information

ESM 1 (PDF 123 kb)

## Data Availability

The datasets generated during and/or analyzed during the current study are available from the corresponding author on reasonable request.
