# Supplementary material for: |Isolation and characterization of novel bacteriophages as a potential therapeutic option for Escherichia coli urinary tract infections
Source: Appl Microbiol Biotechnol. 2021 Jul 12;105(13):5617–29. doi: 10.1007/s00253-021-11432-6 (PMC8285336; doi:10.1007/s00253-021-11432-6)

Applied Microbiology and Biotechnology

Isolation and characterization of novel bacteriophages as a potential therapeutic option for *Escherichia coli* urinary tract infections

Edgar González-Villalobos[a,b,c,d], Rosa María Ribas-Aparicio[a], Gerardo Erbey Rodea Montealegre[b,c], Laura Belmont-Monroy[a,b,c], Yerisaidy Ortega-García[a,b,c], Gerardo Aparicio-Ozores[a], José Luis Balcázar[d,e], Carlos Alberto Eslava-Campos[b,c], Ulises Hernández-Chiñas[b,c], José Molina-López[b,c]*

[a] Departamento de Microbiología, Escuela Nacional de Ciencias Biológicas (ENCB), Instituto Politécnico Nacional (IPN). Prolongación de Carpio y Plan de Ayala s/n, Colonia Santo Tomás, C.P. 11340, Mexico City, Mexico.

[b] Unidad Periférica de Investigación Básica y Clínica en Enfermedades Infecciosas; Departamento de Salud Pública/División de Investigación, Facultad de Medicina, UNAM, C.P. 04510. Mexico City, Mexico.

[c] Laboratorio de Patogenicidad Bacteriana, Unidad de Hemato-Oncología e Investigación. Hospital Infantil de México Federico Gómez/Facultad de Medicina UNAM, C.P. 06720 Mexico City, Mexico.

[d] Catalan Institute for Water Research (ICRA). Girona 17003, Spain.

[e] University of Girona. Girona 17004, Spain.

*Corresponding author:
Dr. José Molina-López
Phone: +52 55 52 28 99 17.
E-mail: joseml@unam.mx

Supplementary material

Supplementary Table S1. Mass spectrometry data from ϕEc3 and ϕEc4 phages. In both cases, similar proteins were detected by mass spectrometry. ID denotes the reference of the identified proteins. M/Z indicates the mass-charge ratio. Peptide refers to a protein sequence.

ID	Coverage	M/Z	Peptide
RNA2 polyprotein	6.5%	871.257	DMISDFK
		887.239	ISTSVAPGR
		1364.076	WITAGLVMPTFK
		1758.347	ISTSVAPGRAQWISER
		1792.164	MFSWKWGSGPIDNPEK
		2383.480	ITASADPVYTLSVPHWLIHHK
Endolysin	19.0%	862.116	QAEDMLR
		1046.608	SGDNLTKIAK
		1347.711	SGDTLSKIAVDNK
		2086.956	QISQAGINLIKSFEGLQLK

Supplementary Fig. S1. Analysis of ϕEc3 and ϕEc4 protein profiles (15% SDS-PAGE with silver staining). ϕEc3, two proteins of 37 kDa and 11 kDa were identified. ϕEc4, two proteins of 37 kDa and 13 kDa were identified.

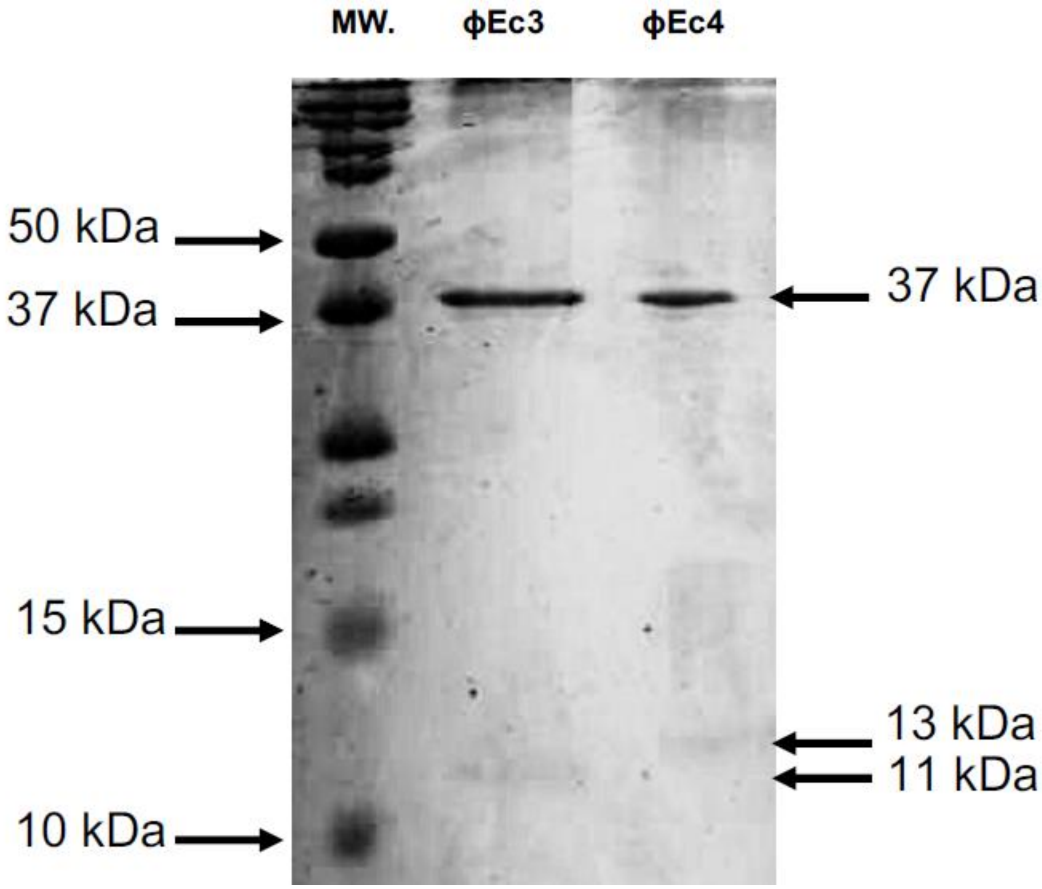

Supplement: Supplementary file 1 — (PDF 123 kb) [file 253_2021_11432_MOESM1_ESM.pdf]
